# Antibiotic resistance and genotype of beta-lactamase producing Escherichia *coli* in nosocomial infections in Cotonou, Benin

**DOI:** 10.1186/s12941-014-0061-1

**Published:** 2015-01-17

**Authors:** Eugénie Anago, Lucie Ayi-Fanou, Casimir D Akpovi, Wilfried B Hounkpe, Micheline Agassounon-Djikpo Tchibozo, Honoré S Bankole, Ambaliou Sanni

**Affiliations:** Laboratoire de Biochimie et de Biologie Moléculaire, Institut des Sciences Biomédicales Appliquées, 03 BP 0420 Cotonou, Bénin; Laboratoire de Recherche en Biologie Appliquée (LARBA), Ecole Polytechnique d’Abomey-Calavi, Université d’Abomey-Calavi, B.P. 2009 Cotonou, Bénin; Laboratoire de Génétique et des Biotechnologies, Faculté des Sciences et Techniques (FAST), Université d’Abomey-Calavi (UAC), 01 BP 1636 RP Cotonou, Bénin

**Keywords:** *Escherichia coli*, ESBL, Resistance gene

## Abstract

**Background:**

Beta lactams are the most commonly used group of antimicrobials worldwide.

The presence of extended-spectrum lactamases (ESBL) affects significantly the treatment of infections due to multidrug resistant strains of gram-negative bacilli. The aim of this study was to characterize the beta-lactamase resistance genes in *Escherichia coli* isolated from nosocomial infections in Cotonou, Benin*.*

**Methods:**

*Escherichia coli* strains were isolated from various biological samples such as urine, pus, vaginal swab, sperm, blood, spinal fluid and catheter. Isolated bacteria were submitted to eleven usual antibiotics, using disc diffusion method according to NCCLS criteria, for resistance analysis. Beta-lactamase production was determined by an acidimetric method with benzylpenicillin. Microbiological characterization of ESBL enzymes was done by double disc synergy test and the resistance genes TEM and SHV were screened by specific PCR.

**Results:**

ESBL phenotype was detected in 29 isolates (35.5%). The most active antibiotic was imipenem (96.4% as susceptibility rate) followed by ceftriaxone (58.3%) and gentamicin (54.8%). High resistance rates were observed with amoxicillin (92.8%), ampicillin (94%) and trimethoprim/sulfamethoxazole (85.7%). The genotype TEM was predominant in ESBL and non ESBL isolates with respectively 72.4% and 80%. SHV-type beta-lactamase genes occurred in 24.1% ESBL strains and in 18.1% of non ESBL isolates.

**Conclusion:**

This study revealed the presence of ESBL producing *Eschericiha coli* in Cotonou. It demonstrated also high resistance rate to antibiotics commonly used for infections treatment. Continuous monitoring and judicious antibiotic usage are required.

## Background

Antibiotics resistance is a paramount issue in medical practice. Production of β-lactamases is an important means by which Gram-negative bacteria exhibit resistance to β-lactam antibiotics [1]. The increasing prevalence of pathogens producing extended-spectrum-lactamases (ESBLs) is showed worldwide in hospitalized patients as well as in out-patients. Infections with ESBL producing organisms are associated with higher rates of mortality, morbidity and healthcare expenditure. ESBLs are often plasmid mediated; though they occur predominantly in *Escherichia coli* and *Klebsiella* species, they have also been described in other genera of the Enterobacteriacea [1,2].

Multidrug resistance in ESBL producers limit therapeutic options and subsequently facilitate the dissemination of these bacteria strains. Several studies have reported the increasing resistance rate of commonly prescribed antibiotics such as ampicillin, trimethoprim/sulfamethoxazole and ciprofloxacin in clinical isolates of *Escherichia coli* [3-6].

Antibiotic resistance varies according to geographic locations and is directly proportional to the use and misuse of antibiotics. Understanding the effect of drug resistance is crucial because of its deep impact on the treatment of infections. In Benin, little information about resistance to third generation cephalosporin as well as multi drug resistance in *Escherichia coli* is known*.* As antibiotic treatment must rely on antimicrobial susceptible pattern, current knowledge on susceptibility is essential for appropriate therapy. Previous studies reported the presence of TEM and SHV in nosocomial and community isolated *Escherichia coli* strains in Benin [7,8].

The aim of the present study was to characterize clinical isolates of *Escherichia coli* obtained from several infections in Cotonou in order to (i) determine the susceptibility patterns to antibiotics, (ii) evaluate the prevalence of ESBL and (iii) identify the genes involved in the resistance.

## Materials and methods

### Bacterial strains

The study was carried out in three hospitals in Cotonou, from September 2012 to April 2013. Consecutive, non-repeated nosocomial *Escherichia coli* isolates obtained from urine, pus, vaginal swab, sperm, blood, spinal fluid and catheter samples received in the bacteriology laboratories were analyzed. The isolated microorganisms were considered as nosocomial origin if they were isolated from patients admitted to the hospitals since 48 hours or more. The main pathogens were identified by cultural characteristics and standard biochemical procedures and were confirmed with API 20 E (Biomérieux, Marcy l’Etoile, France) identification system.

### Antibiotic susceptibility testing and detection of ESBL

Antibiotic susceptibility was performed by the disc diffusion method on Mueller Hinton agar (Bio-Rad, Marne la Coquette, France) according to the recommendations of the Antibiogram Committee of the French Society for Microbiology (Comité de l’Antibiogramme de la Société Française de Microbiologie) [9]. The following antibiotic discs (drug concentration in μg) were tested: amoxicillin (25 μg), amoxicillin/clavulanic acid (20/10 μg), ampicillin (10 μg), imipenem (10 μg), cefotaxime (30 μg), ceftriaxone (30 μg), ciprofloxacin (5 μg), norfloxacin (5 μg), amikacin (30 μg), gentamicin (15 μg) and trimethoprim/ sulfamethoxazole (1.25/23.75 μg), all from Bio-Rad (Bio-Rad, Marne la Coquette, France).

ESBL phenotypes were detected by double-disk synergy according to the method described by Jarlier *et al.* [10]. Disks of cefotaxime and ceftriaxone were placed 20 mm from an amoxicillin/clavulanate disk. Enhancement of the inhibition zone of the third-generation cephalosporin toward the amoxicillin/clavulanate disk indicated the possible presence of an ESBL. *Escherichia coli* ATCC 25922 was used as control.

### Biochemical detection of beta-lactamase production

The presence of beta-lactamase was tested by an acidimetric method using benzylpenicillin as substrate [11]. A single colony was resuspended and mixed with the indicator solution. The indicator solution was prepared by adding 1 ml of sterile distilled water and 100 μl of 1% phenol red solution to a vial of one million units of sodium benzylpenicillin (Crystapen, Glaxo). A solution of 1 N sodium hydroxide was added until the development of violet color (pH 8.5). Several colonies were suspended in NaCl, 9‰ to get a dense suspension. 150 μl of penicillin phenol red solution was added and the color development observed within 1 hour. The solution turned yellow in the presence of beta-lactamase.

### Detection of beta-lactamase genes

All the strains were further analyzed by PCR to detect beta-lactamase genes. Total DNA extraction was performed using the heat-shock method. Briefly, a single bacteria colony was inoculated into 5 ml of Luria-Bertani broth (Biorad, Marne la Coquette, France) and incubated for 20 h at 37°C. Cells from 1.5 ml of the overnight culture were harvested by centrifugation at 7000 RPM for 5 min. After the supernatant was removed, the pellet was washed twice with sterile water and resuspended in 500 μl of sterile water. This suspension was incubated at 95°C during 10 min. The supernatant was stored at −20°C for PCR analysis.

The presence of beta-lactamase genes, *bla*_TEM_ and *bla*_SHV_, was detected using specific primers: for the TEM genes OT-1-F [5′-TTGGGTGCACGAGTGGG TTA-3′] and OT-2-R [5′-TAATTGTTGCCGGGAAGCTA-3′] which amplified a 465 bp fragment [12]; for the SHV genes, SHV-A[5′-CACTCAAGGATGTATTGTG-3′] and SHV-B[5′-TTAGCGTTGCCAGTGCTCG-3′] which amplified a fragment of 885 bp [13].

Amplification reactions were performed in a volume of 30 μl containing 4 μl of supernatant, (volume) PCR buffer (1x), MgCl_2_ (1.5 mM), (volume) dNTPs (200 μM), (volume) primer (0.5 mM), 0.2 μL of Taq DNA polymerase (1,5U). An Biometra thermal cycler was used for the amplification. The cycling conditions were the following:

TEM: initial denaturation at 94°C for 5 min, followed by 30 cycles of denaturation at 94°C for 30 s, annealing at 52°C for 30 s , extension at 72°C for 1 min;

SHV: initial denaturation at 96°C for 15 s, followed by 30 cycles of denaturation at 96°C for 15 s, annealing at 50°C for 15 s, extension at 72°C for 2 min. Both PCR programs were followed by a final extension step of 10 min.

Amplified PCR products were separated on 1.5% agarose gels, stained with ethidium bromide and visualized under UV illumination.

### Statistical analysis

Data were analyzed with Epi Info® version 3.5.4. Differences in antibiotic susceptibility among different groups were statistically analyzed by the Fisher exact test. An associated P*-value* < 0.05 was considered significant.

## Results

A total of 84 nosocomial *Escherichia coli* isolates were included in this study. The majority of isolates (72 strains: 85.7%) were from urine samples. Specimens from pus represented 4.76% of the isolates, followed by vagina swab and spinal liquid with 2.38% each. Isolates from sperm and catheter were 1.2% each.

The antibiotics susceptibility test revealed that the most efficient antibiotics were imipenem (96.4% as susceptibility rate) followed by ceftriaxone (58.3%) and gentamicin (54.8%). High resistance rates were observed with amoxicillin (92.8%), ampicillin (94%), and trimethoprim/sulfamethoxazole (85.7%). Resistance to amoxicillin/clavulanic acid was 85.7% with a high rate of intermediate resistance (46.7%). We observed homogeneity in the resistance profile of ESBL-producers which were multi drugs resistant with at least a resistance to 8 antibiotics out of the 11 tested (Table 1).

**Table 1:** **Antibiotic susceptibility pattern of ESBL and non ESBL producing isolates**

**Antibiotics**	**Susceptibility rate %**		**ESBL (n = 29)**	**Non-ESBL (n = 55)**	p-value*
	**R**	**S**			
AM	97.6	2.4	29 (100%)	50 (91 %)	0.4
AMC	85.7	14.3	24 (82.7%)	15 (27.3%)	10^−4^
AMX	95.2	4.8	29 (100%)	49 (89.1%)	0.17
IPM	3.6	96.4	01 (3.4%)	00 (0%)	0.3
CTX	56.5	40.5	28 (96.5%)	01 (1.8%)	<10^−9^
CRO	44.7	58.3	28 (96.5%)	01 (1.8%)	10^−3^
CIP	91.7	8.3	29 (100%)	14 (25.5%)	10^−10^
NOR	53.6	46.4	28 (96.5%)	14 (25.5%)	<10^−9^
AN	83.3	16.7	21(72.4%)	19 (34.5%)	0.04
G	45.2	54.8	24 (82.7)	12 (21.8)	10^−7^
SXT	86.9	13.1	29 (100%)	43 (78.2%)	6.10^−3^

*P value is for the comparison of resistance of ESBL-producers with non-producers.

AM: Ampicilline; AMC: Amoxicilline/clavulanic acid; AMX: Amoxicilline; IPM: Imipenem; CTX: Cefotaxime; CRO: Ceftriaxone; CIP: Ciprofloxacine; NOR: Norfloxacine; AN: Amikacine; G: Gentamicine; SXT: Trimethoprim/Sulfamethoxazole.

According to the biochemical detection of beta-lactamase production, 87.0% of isolates were positive using the acidimetric test by hydrolyzing penicillin G.

Of the 84 isolates screened for ESBL production by the double disk test, 29 (35.5%) were positive using either cefotaxime or ceftriaxone. Among these ESBL-producers, 21 were from urinary tract infection (UTI) and 8 from other infection sites. The other infection sites seem to be disproportionally represented than UTI (27.6% versus 14%). This fact could be explained with reduced number of samples drawn from non-urinary infections.

Comparison of susceptibility rates of ESBL producers with those of ESBL non producers showed similar values for amoxicillin, ampicillin and imipenem. No statistically significant difference between the two groups was observed (Table 1). For all other tested antibiotics, ESBL strains were more resistant than non ESBL strains.

According to the PCR, the genotypes TEM and SHV were distributed as follow: bla_TEM_ 65 (77.4%) and bla_SHV_ 17 (20.2%). These genotypes occurred singularly in 54 isolates (64.3%) with 51(94.4%) for bla_TEM_ and 3 (5.6%) for bla_SHV_. Both TEM and SHV genes were present in 14 isolates (16.7%) and absent in 16 isolates (19%) including 10 non ESBL strains and 6 ESBL strains.

**Figure 1:**
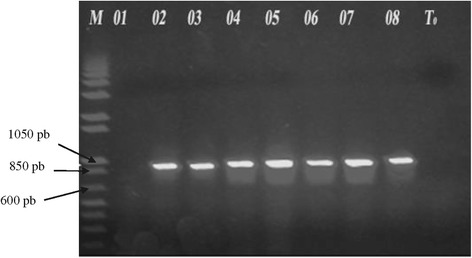
**Agarose gel of PCR products following amplification of SHV genes.** M: Molecular weight marker; Lanes 02 to 08: SHV positive samples; T_0_: negative probe without DNA.

Out of the 29 isolates with ESBL phenotype, the PCR analysis revealed 72.4% strains with the genotype TEM, 24.1% with the genotype SHV and 5 strains harbored both genes. Figure 1 showed the agarose gel of PCR products following amplification of SHV genes.

As shown in Table 2, there were no difference in resistance rate against ceftriaxone and cefotaxime. No difference was found between the inhibition diameters of both antibiotics regarding the genotypes TEM and SHV.

**Table 2:** **Association between ESBL genotype and antibiotic sensibility profile**

**Genotypes**	**Presence**	**Cefotaxime ceftriaxone n (%)**			**Fisher’s exact test**
		**S**	**I**	**R**	***p-value***
TEM	+	0 (0%)	0 (0%)	21 (100%)	0.27
	-	0 (0%)	1 (12.5%)	7 (87.5%)	
SHV	+	0 (0%)	0 (0%)	7 (100%)	0.76
	-	0 (0%)	1 (4.5%)	21 (95.5%)	
TEM + SHV	+	0 (0%)	0 (0%)	5 (100%)	0.54
	-	0 (0%)	1 (16.7%)	5 (83.3%)	

S = sensitive; I = intermediate; R = resistant. + indicate the presence of the genotype. – indicate the absence of the genotype.

## Discussion

Our study was carried out in three hospitals in Cotonou, Benin. Of the 84 isolates tested, 72 (85.7%) were from urinary tract infections (UTI). This finding is similar to results previously described by several studies [7,14-16]. This was expected since clinical isolates of *Escherichia coli* were predominantly responsible of UTI [17,18].

The highest susceptibility of *E. coli* isolates was found to imipenem (96.4%). In 2007 we have showed that the resistance rate to imipenem was 5% in ESBL producers and 2% in non ESBL isolates [7]. A similar high sensibility (93%) was observed by Muvunyi *et al.* in Rwanda [19]. Studies conducted in Iran [20], Morocco [21] and Nigeria [22] reported almost high sensibility of *E. coli* isolates to imipenem at rates of 98.4%, 96.7% and 92.5% respectively. This finding may be due to the stability and the high activity of carbapenem against most beta-lactamases.

High resistance rates were founded to commonly used antibiotics in Benin such as ampicillin (97.6%), amoxicillin (95.2%) and trimethoprim/sulfamethoxazole (86.9%). The observed high rates may be due to uncontrolled consumption, consequence of easy access to inefficient and cheap antibiotics.

Among the tested fluoroquinolones, only 8.3% of the isolates were sensitive to ciprofloxacin. Norfloxacin was more efficient with 44.6% of sensitive strains, but lower than the result reported by Thakur *et al.* [6] with 52.5% of sensitivity. Previous studies in Benin reported lower resistance rates of ciprofloxacin compared to our study. Ahoyo *et al*. reported 48% and 16% resistance to ciprofloxacin in ESBL and non ESBL *E. coli* from nosocomial infections, respectively [7]. Our survey realized in 2004 showed resistance rate of 18% in ESBL producers and 14% in non ESBL isolates from community acquired UTI [8]. The higher rate of resistance to ciprofloxacin in the current study could be explained by intensive use of ciprofloxacin in the past decade.

Lower resistance rates to ciprofloxacin, usually prescribed in uncomplicated UTI and other infections, have been reported in Nepal (45%) [6], in India (52.80%) [23] and in Iran (60%) [20]. It was showed that uncontrolled use of quinolones in the past years led to a growing resistance to these antibiotics particularly to ciprofloxacin worldwide [24,25]. The use of ciprofloxacin selected also ESBL producers due to the widespread co-detection of both resistance mechanisms [26,27].

High resistance rate to amoxicillin/clavulanate acid was also observed (85.7%). This finding is in agreement with previous investigations in Benin. In 2007, we reported resistance rates of 97.5% in ESBL and 53% in non ESBL *E. coli* strains from nosocomial infections [7]. In our previous study performed in 2004, we detected 72% of resistance in ESBL and 9% in non ESBL *E. coli* isolates from out-patients [8].

We showed in this study that 87% of isolated strains were positive for beta-lactamase detection by acidimetric method indicating that the most important mechanism of resistance to beta-lactam antibiotics is the production of beta-lactamase.

The high prevalence of ESBL-producing strains (35.5%) was similar to those generally shown in developing countries.

Our finding is very close to the prevalence ESBL reported in India (35.45%) [28] and similar to the prevalence in Tanzania (39.1%) [29]. Previous studies in Benin revealed relative high prevalence rates of ESBL producing *E. coli*. In 2007, Ahoyo *et al*. showed a prevalence of 22% in isolates from various nosocomial infections. In 2004, we founded that 14.8% of *E. coli* strains isolated from urinary tract infection (UTI) were ESBL [8]. Lower ESBL prevalence was described in Morocco (1.3%) in UTI isolated strains [30], in Cameroon (16%) in strains isolated from feces in the community [25].

Among the ESBL-producers, the other infection sites seem to be disproportionally represented than UTI (27.6% versus 14%). This fact could be explained with a small number of samples drawn from non-urinary infections.

The comparison between ESBL producing strains and non ESBL showed that ESBL-producers were significantly more resistant to cephalosporins, quinolones, aminosides, trimethoprim/sulfamethoxazole and amoxicillin/clavulanic acid than non-ESBL producers. The genes encoding ESBLs are usually located in transferable plasmids that may also carry other resistance determinants, such as those for resistance to aminoglycosides, tetracyclines, chloramphenicol, trimethoprim, sulphamides, and quinolones [31,32].

The genotype TEM was predominant in ESBL and non ESBL isolates with respectively 72,4% and 80%. This fact was generally observed in *E. coli*. The remaining 6 ESBL strains which were non TEM and non SHV could harbor CTX-M genes. Widespread dissemination of these genes has been described in Africa and elsewhere [14,25,31].

Out of the 65 strains harboring TEM genes, 59 showed positive result for the detection of enzyme production using hydrolysis of penicillin. Of the 17 SHV positive strains, 15 (80.95%) were positive for this test.

In this study, the phenotypic screening for ESBL was realized by resistance to cefotaxime or ceftriaxone. The presence of genotype TEM and SHV could not predict the resistance pattern to these cephalosporins. Our finding is consistent with the results reported by Maina *et al.* who found no significant association between genotypes TEM and SHV and susceptibility to cefotaxime and ceftriaxone [33].

## Conclusions

This study reveals the presence of ESBL producing *Escherichia coli* in clinical isolates from several hospitals in Cotonou. The use of some first line treatment antibiotics such as penicillin and trimethoprim/sulfamethoxazole seems inappropriate. Antibiotics resistance surveillance and the determination of molecular characteristics of ESBL isolates are primordial to ensure the judicious use of antimicrobial drugs.

## References

[1] Bradford PA (2001). Extended-spectrum beta-lactamases in the 21st century: characterization, epidemiology, and detection of this important resistance threat. Clin Microbiol Rev.

[2] Paterson DL, Bonomo RA (2005). Extended-spectrum beta-lactamases: a clinical update. Clin Microbiol Rev.

[3] Bean DC, Krahe D, Wareham D (2008). Antimicrobial resistance in community and nosocomial *Escherichia coli* urinary tract isolates. Ann Clin Microbiol Antimicrob.

[4] Dromigny JA, Nabeth P, Juergens-Behr A, Perrier-Gros JD (2005). Risk factor for antibiotic-resistant *Escherichia coli* isolated from community-acquired urinary tract infections in Dakar, Sénégal. J Antimicrobial Chemother.

[5] Drago L, Nicola L, Mattina R, De Vecchi E (2010). In vitro selection of resistance in *Escherichia coli* and *Klebsiella* spp. at in vivo fluoroquinolone concentrations. BMC Microbiol.

[6] Thakur P, Ghimire P, Rijal KR, Singh GK (2012). Antimicrobial resistance pattern of *Escherichia coli* isolated from urine samples in patients visiting tertiary health care centre in eastern Nepal. Sunsari Tech Coll J.

[7] Ahoyo AT, Baba-Moussa L, Anago AE, Avogbe P, Missihoun TD, Loko F (2007). Incidence d’infections liées à *Escherichia coli* producteur de bêta-lactamase à spectre élargi au Centre hospitalier départemental du Zou et Collines au Bénin. Med Mal Infect.

[8] ANAGO E. Activités antibactériennes de quelques plantes de la pharmacopée africaine sur des souches de *Escherichia coli* productrices de bêta-lactamases*.* PhD thesis. In: CID CAMES, editor. Ouagadougou: Université d’Abomey-Calavi, Faculté des Sciences et Techniques, Ecole doctorale Sciences de la vie; 2009.

[9] Comité de l’Antibiogramme de la Société Française de Microbiologie, Recommandations 2012. Société Française de Microbiology, editor. Paris Cedex; 2012. p.5-23.

[10] Jarlier V, Nicolas MH, Fournier G, Philipon A (1988). Extended broad-spectrum β-lactamases conferring transferable resistance to newer β-lactam agent in *Enterobacteriaceae*: hospital prevalence and susceptibility pattern. Rev Infect Dis.

[11] Koneman EW. Test for determining inhibitory. In: Koneman’s color atlas and textbook of diagnostic microbiology. 5th ed. Lippincott Williams and Wilkins, editors. Philadelphia. 2006. p. 1001.

[12] Arlet G, Philippon A (1991). Construction by polymerase chain reaction and intragenic DNA probes for three main types of transferable β-lactamases (TEM, SHV, CARB). FEMS Microbiol Lett.

[13] Pitout JDD, Thomson KS, Hanson ND, Ehrhardt AF, Moland ES, Sanders CC (1998). β-lactamases responsible for resistance to expanded-spectrum cephalosporins in *Klebsiella pneumoniae*, *Escherichia coli* and *Proteus mirabilis* isolates recovered in South Africa. Antimicrob Agents Chemother.

[14] Iroha IR, Esimone CO, Neumann S, Marlinghaus L, Korte M, Szabados F (2012). First description of Escherichia coli producing CTX-M-15- extended spectrum beta lactamase (ESBL) in out-patients from south eastern Nigeria. Ann Clin Microbiol Antimicrob.

[15] Park YS, Adams-Haduch JM, Shutt KA, Yarabinec DM, Johnson LE, Hingwe (2012). High prevalence of extended-spectrum beta-lactamase-producing pathogens: results of a surveillance study in two hospitals in Ujjain, India. Infect Drug Resist.

[16] Oberoi L, Singh N, Sharma P, Aggarwal A (2013). ESBL, MBL and Ampc β lactamases producing superbugs – Havoc in the Intensive Care Units of Punjab India. J Clin Diagn Res.

[17] Eyquem A, Alouf J, Montagnier L (2000). Etat actuel de la sensibilité des bactéries aux antibiotiques en France, en pratique de ville et en pratique hospitalière. Traité de microbiologie clinique.

[18] Gupta K (2002). Addressing antibiotic resistance. Am J Med.

[19] Muvunyi CM, Masaisa F, Bayingana C, Mutesa L, Musemakweri A, Muhirwa G (2011). Decreased susceptibility to commonly used antimicrobial agents in bacterial pathogens isolated from urinary tract infections in Rwanda: need for new antimicrobial guidelines. Am J Trop Med Hyg.

[20] Mohammedi-Mehr M, Feizabadi MM (2011). Antimicrobial resistance pattern of Gram-negative bacilli isolated from patients at ICUs of Army hospitals in Iran. Iranian J Microbiol.

[21] Mohammad-Jafari H, Saffar MJ, Nemate I, Saffar H, Khalilian AR (2012). Increasing antibiotic resistance among uropathogens isolated during years 2006–2009:impact on the empirical management. Int Braz J Urol.

[22] Ejikeugwu PC, Ugwu CM, Araka CO, Gugu TH, Iroha IR, Adikwu MU (2012). Imipenem and meropenem resistance amongst ESBL producing Escherichia coli and Klebsiella pneumonia clinical isolates. Int Res J Microbiol.

[23] Rashid M, Modi S, Shukla I, Chander Y (2013). Prevalence and antibiogram of extended spectrum betalactamase producing *Escherichia coli*. J Evol Med Dent Sci.

[24] Drago L, Nicola L, Mattina R, De Vecchi E (2010). In vitro selection of resistance in *Escherichia coli* and *Klebsiella spp*. at in vivo fluoroquinolone concentrations. BMC Microbiol.

[25] Lonchel CM, Meex C, Gangoue-Pieboji J, Boreux R, Assoumou MC, Melin P (2012). Proportion of extended-spectrum β-lactamase-producing Enterobacteriaceae in community setting in Ngaoundere, Cameroon. BMC Infect Dis.

[26] Schaumburg F, Alabi A, Kokou C, Grobusch MP, Köck R, Kaba H (2013). High burden of extended-spectrum beta-lactamase-producing Enterobacteriaceae in Gabon. J Antimicrob Chemother.

[27] Martinez-Martinez L, Pascual A, Jacoby GA (1998). Quinolone resistance from a transferable plasmid. Lancet.

[28] Kashyap G, Gupta S, Mamoria VP, Durlabhji P, Jain D (2013). Increasing prevalence of extended spectrum beta lactamases (ESBL) producing E. coli and Klebsiella spp in Outpatient Departements (OPDs) patients in urinary tract infections (UTIs) in tertiary care hospital. Int J Cur Res Rev.

[29] Moyo SJ, Aboud S, Kasubi M, Lyamuya EF, Maselle SY (2010). Antimicrobial resistance among producers and non-producers of extended spectrum beta-lactamases in urinary isolates at a tertiary Hospital in Tanzania. BMC Res Notes.

[30] Bourjilat F, Bouchrif B, Dersi N, Claude JD, Amarouch H, Timinouni M (2011). Emergence of extended-spectrum beta-lactamases-producing Escherichia coli in community-acquired urinary infections in Casablanca, Morocco. J Infect Dev Ctries.

[31] Jacoby GA, Sutton L (1991). Properties of plasmids responsible for production of extended-spectrum betalactamases. Antimicrob Agents Chemother.

[32] Philippon A, Arlet G, Lagrange PH (1994). Origin and impact of plasmid-mediated extended-spectrum beta-lactamases. Eur J Clin Microbiol Infect Dis.

[33] Maina D, Revathi G, Kariuki S, Ozwara H (2012). Genotypes and cephalosporin susceptibility in extended-spectrum beta-lactamase producing enterobacteriaceae in the community. J Infect Dev Ctries.

